# Impact of cardiovascular magnetic resonance on management and clinical decision-making in patients presenting with chest pain, elevated troponin and unobstructed coronary artery

**DOI:** 10.1186/1532-429X-18-S1-P118

**Published:** 2016-01-27

**Authors:** Amardeep Ghosh Dastidar, Estefania De Garate, Jonathan C Rodrigues, Anna Baritussio, Alessandra Scatteia, Priyanka Singhal, Andreas Baumbach, Angus K Nightingale, Chiara Bucciarelli-Ducci

**Affiliations:** NIHR Bristol Cardiovascular Biomedical Research Unit, Bristol Heart Institute, Bristol, UK

## Background

Management of patients presenting with chest pain, elevated troponin and unobstructed coronary artery is challenging. Cardiovascular magnetic resonance (CMR) can provide important diagnostic and prognostic information in this cohort. However, the evidence of impact of CMR on clinical management is lacking. We sought to evaluate the impact of CMR on diagnosis and clinical management in patients with chest pain, elevated troponin and unobstructed coronaries.

## Methods

We studied consecutive patients presenting with chest pain, elevated troponin and unobstructed coronaries referred for CMR at a large tertiary cardiothoracic centre. Definitions for "significant clinical impact" of CMR were pre-defined and collected directly from medical records. Categories of significant clinical impact included: change in the pre-CMR diagnosis, medication change, hospital discharge, as well as performance or avoidance of invasive procedures (device therapy, angiography or myocardial biopsy). A comprehensive CMR protocol was used including long and short axis cines, T2 weighted oedema sequences and early and late gadolinium enhancement imaging.

## Results

204 consecutive patients (mean age 55 yrs) were included (51% males). A cause for the troponin rise was found in 70% of patients. Overall, CMR had a significant clinical impact in 66% of patients, with CMR leading to change in the final diagnosis in 54% of cases. CMR results directly led to performance of invasive procedures in 5%. In a multivariable model that included clinical and imaging parameters, presence of late gadolinium enhancement (LGE) and age were the only independent predictors of "significant clinical impact" (LGE OR 2.3, 95% CI 1.12-4.74, p = 0.02, & Age OR 1.03, 95% CI 1.01-1.058, p = 0.001)

## Conclusions

CMR made a significant additive clinical impact on management, decision-making and diagnosis in 66% of patients with chest pain, elevated troponin and unobstructed coronaries. This additive impact was seen despite use of prior echocardiography, troponin assay and coronary angiogram in this patient group. The presence of LGE was the best independent predictor of significant clinical impact following CMR.

**Table 1:** Multivariate analysis of predictors of clinical impact

Predictor of clinical impact	Sig.	OR	95% Conf interval	
			Lower	Upper
Age	.001	1.035	1.014	1.058
Sex	.510	.791	.393	1.591
Troponin I	.523	1.000	1.000	1.001
iEDV	.295	1.008	.993	1.024
LVEF	.947	1.001	.971	1.033
RWMA	.949	.975	.451	2.110
Oedema	.497	1.325	.589	2.982
LGE	.023	2.306	1.121	4.744

Variables: Age, Sex, Troponin I, iEDV- indexed end diastolic volume, LVEF-left ventricular ejection fraction, RWMA - regional wall motion abnormality, Myocardial Oedema on T2 weighted imaging, LGE - late gadolinium enhancement

